# Influence of Long-Term Dietary Cobalt Supplementation on Lactation Performance and Reproductive Efficiency in Maghrabi She-Camels

**DOI:** 10.1007/s12011-025-04551-y

**Published:** 2025-03-03

**Authors:** Mohamed S. Ayyat, Usama M. Abdel Monem, Tarek H. Mostafa, Rafiq M. Thabet, Khaled M. Abd El-Latif, Adham A. Al-Sagheer

**Affiliations:** 1 Department of Animal Production, Faculty of Agriculture, Zagazig University, Zagazig, Egypt; 2 Camel Research Department, Animal Production Research Institute, Giza, Dokki Egypt; 3 Specialized Hospital, Ain Shams University, Cairo, Egypt

**Keywords:** Cobalt glucoheptonate, Dromedary camel, Milk yield, Reproductive performance, Blood biochemistry, Camel calves

## Abstract

This study examined the effects of cobalt supplementation, given from three months prepartum to nine months postpartum, on lactation performance, reproductive efficiency, blood biochemistry, colostrum immunoglobulin levels, and hormonal profiles in she-camels, along with the growth of their calves. Twenty Maghrabi she-camels, weighing 489 ± 2.31 kg and aged 6–9 years, were used in this study. The camels were divided into four groups: one control group fed only the basal diet, and three treatment groups receiving 0.16, 0.32, and 0.48 mg of cobalt per kg of feed as cobalt glucoheptonate. Results indicated that cobalt supplementation significantly increased colostrum immunoglobulin concentrations (IgG, IgM, and IgA), as well as monthly milk yield and lactation period. Milk contents of total solids, protein, and lactose also improved in the supplemented groups, particularly at 0.32 mg/kg cobalt. Additionally, pre- and post-partum serum cobalt, vitamin B_12_, T3, T4, progesterone, and estradiol levels were higher in the cobalt-treated groups. Enhanced reproductive outcomes included shorter postpartum intervals to first estrus, reduced services per conception, and improved weaning weights and average daily gains of calves in all cobalt-supplemented groups. In conclusion, dietary cobalt supplementation at a level of 0.32 mg/kg from three months prepartum to nine months postpartum significantly enhanced the lactation and reproductive performance of Maghrabi she-camels, increased colostrum immunoglobulin levels, and positively affected the growth performance of their calves.

## Introduction

Sustainability in the livestock industry is becoming essential, especially in dry countries like North Africa, where resource limitations hinder conventional farming methods [1]. The one-humped camels are an essential part of the agricultural landscape, supplying milk, meat, and transportation in challenging regions [2]. The camel is well adapted to arid environments, functioning as an environmentally friendly animal. Additionally, camel farming represents a low environmental pressure activity. The camel is adapted to thrive in harsh conditions where other animals may not survive [3]. Dromedary camels, characterized by induced ovulation and seasonal activity, are prevalent in the North African region, specifically Maghrebi camels, which have medium-sized bodies, small pointed humps, and relatively low milk production [4]. Enhancing she-camel productivity through nutritional supplementation, particularly with trace minerals, is a promising approach for boosting both economic value and sustainability [5, 6]. Targeted dietary strategies provide effective methods to enhance health and overall productivity in camel farming [7].

Cobalt is an important trace mineral necessary for proper microbial metabolism in the rumen, particularly as an important component in the synthesis of cobalamin (vitamin B_12_) [8]. This vitamin is produced by ruminal microbes in the presence of cobalt [9]. According to several studies, the amount of cobalt in the ration has a direct correlation with the amount of vitamin B_12_ in the cattle's rumen [10, 11]. Vitamin B_12_, synthesized by rumen microbes, is crucial for various important biochemical processes. It promotes glucose generation from ruminal propionate, helps with methionine synthesis, and is essential for DNA methylation, with cobalt serving as a key component in these metabolic pathways [12]. Cobalt carbonate, sulfate, chloride, nitrate, propionate, acetate, and glucoheptonate serve as efficacious cobalt sources for ruminants [13], but they vary in microbial utilization. Cobalt carbonate exhibits low water solubility, whereas acetate and lactate dissolve easily in water, affecting its availability to rumen bacteria [14].

Previous research found that the dietary requirement for cobalt is approximately 0.11 mg/kg dry matter (DM), while current recommendations suggest supplementation up to 0.20 mg cobalt /kg DM, which appears to enhance animal productivity, particularly in dairy ruminants [15]. Higher cobalt levels, reaching up to 0.78 mg/kg DM, can lead to notable enhancements in milk productivity, with milk yield increases of up to 15% [16]. A study indicated that cobalt citrate at doses of 19 mg and 34 mg Co/kg DM led to a 4.5% increase in daily milk production [17]. While cobalt supplementation offers advantages, some studies suggested that excessive amounts may not boost milk production proportionally, highlighting the necessity for balanced supplementation adapted to specific dietary needs and environmental conditions [15]. Although numerous studies have explored cobalt supplementation’s effects on lactation and reproductive outcomes in ruminants, research on camels remains limited.

Given the above data, the study hypothesized that dietary supplementation with cobalt could enhance lactation performance and reproductive efficiency in she-camels, as well as improve the growth performance of their calves. The objective of this study was to evaluate the impact of dietary cobalt supplementation, specifically in the form of cobalt glucoheptonate, on lactation performance, reproductive health, blood biochemical profiles, colostrum immunoglobulin content, and thyroid and ovarian hormone levels in Maghrabi she-camels, along with the growth performance of their calves. The experiment lasted 12 months and included both pre-partum (three months) and post-partum (nine months) periods.

## Materials and Methods

The experiment was conducted at the Marsa Matrouh Station, Center for Studies and Development of Camel Production, located in the Marsa Matrouh Governorate, Egypt, under the supervision of Animal Production Research Institute of the Agricultural Research Center, Giza, Egypt.

### Animal Management and Feeding Protocol

Twenty Maghrabi dromedary she-camels (*Camelus dromedarius*) were selected for the study, aged between 6 and 9 years, weighing from 489 ± 2.31 kg, and at their 2nd or 3rd parity. The animals, with no history of peripartum illnesses, were all in the late stages of pregnancy. Based on their body weight and parity, the camels were randomly allocated into four experimental groups, with five camels in each group. The animals were kept for 14 days as an acclimatization period. The animals were housed in semi-open pens until they were moved to the maternity unit approximately 1–2 days before giving birth. All camels were provided with a similar basal diet, formulated to meet their needs during both pre- and post- partum periods [18, 19]. Before parturition, each animal received 4.5 kg of a concentrate feed mixture (CFM), 2 kg of berseem hay (BH), and 2 kg of rice straw (RS) daily. After parturition, the feeding regimen was adjusted to 3.5 kg CFM, 2.5 kg BH, and 2 kg RS per animal. The diet was formulated to meet the nutritional demands according to the camels’ milk production, weight, and reproductive status. Feed was offered twice a day at 8 a.m. and 5 p.m., with free access to clean water. The CFM was prepared in mash form. For each treatment group, the cobalt supplement was first pre-mixed with 100 g of CFM and then thoroughly blended into the remaining CFM to obtain a uniform and consistent inclusion level across the feed. The chemical composition of the feeds used in the study (Table 1) was analyzed according to AOAC [20].

**Table 1:** Chemical composition of berseem hay, rice straw, and concentrate feed mixture fed to all groups (%, on DM basis)

Item	Berseem hay	Rice straw	Concentrate feed mixture
Dry matter	88.46	88.91	89.44
Crude protein	11.30	2.53	12.24
Ether extract	3.20	1.52	4.64
Crude fiber	30.50	35.69	8.85
Nitrogen free extract	37.70	40.50	66.70
Cobalt (mg/kg)	0.41	0.05	0.23

The concentrate feed mixture was composed of wheat bran (25%), yellow corn (25%), barley (20%), rice bran (15%), cottonseed meal (9%), molasses (3%), premix (2%), and salt (1%)

In the experimental groups, one group used as the control, they only fed the basal diet without any supplementation. The other three groups were provided with the basal diet enhanced with cobalt glucoheptonate (COPRO, Zinpro Corporation, Minnesota, USA) at levels of 0.16, 0.32, and 0.48 mg of cobalt per kg of feed. The selected cobalt concentrations in this study were based on prior research evaluating cobalt supplementation in other lactating ruminants [15, 17]. Pregnancy was confirmed via ultrasound to accurately define the start of cobalt supplementation, which began three months before calving. Calving occurred within a 10-day period, and supplementation continued for nine months postpartum for each animal. During the trial period, all animals were monitored daily for health and welfare. No mortality or major health issues were observed in the in either the she-camels or their calves.

### Body Weight Measurements and Colostrum Analysis

At the beginning of the experimental period (three months pre-natal), the camels in all groups were weighed to determine their initial live body weight (LBW). Afterward, the live body weight of the camels was recorded both during the pre-partum period (three months before calving) and the postpartum period (following calving). Similarly, the calves were weighed immediately after birth and again at 7 months of age (weaning age). From these measurements, the total and average daily weight gain of the calves was calculated. Following parturition, calves were permitted to nurse from their mothers for the first week, after which the dams were moved to the milking unit. Colostrum samples were collected from each dam within an hour of birth (first milking) on days 1, 2, and 3 postnatal for immunoglobulin analysis. The amounts of immunoglobulins (IgG, IgM, and IgA) in colostrum were measured using bovine radial immunodiffusion (RID) kits, according to the manufacturer's protocol (Ltd, Birmingham, UK) following the methods of Fahey and McKelvey [21] and Mancini et al. [22].

### Lactation Performance and Milk Composition

All camels were hand-milked twice daily, at 7 a.m. and 5 p.m. Milk yield was measured by weighing the milk produced after each milking on two consecutive days each week, and monthly milk yield was calculated based on these values. Monthly milk samples (a composite of morning and evening milking) were collected from each animal for the analysis of milk composition and physical characteristics. Total solids, protein, fat, lactose, and ash content were analyzed using a MilkoScan (Model 133 B, Rajasthan Electronics & Instruments Limited, Rajasthan, India). The yields of milk protein, lactose, total solids, and solids-not-fat were calculated by multiplying the total milk yield by the respective percentage composition of each component.

For cobalt analysis, during the third month of lactation, 30 ml milk samples were collected from each camel (a composite of morning and evening milking) into clean glass bottles. The samples were immediately frozen at − 20˚C until analysis. Milk samples were digested with nitric acid hydrogen peroxide, heated to 120 °C until brown fumes appeared. The resulting solution was cooled, filtered into a 25 ml volumetric flask, and diluted with deionized water. Then samples were analyzed for cobalt content using atomic absorption spectrophotometer [23].

### Blood Sample Collection and Analysis

Blood samples were drawn from all camels in each group one month prior to parturition and one month following parturition. Blood samples were collected before the morning feeding. Samples were collected from the jugular vein into heparinized vacuum tubes (Kuwait Egypt for Medical Industries Co., El Obour City, Egypt). Plasma was isolated by centrifugation of the blood at 4000 rpm for 20 min and preserved at −20 °C until subsequent analysis. The concentrations of triglycerides [24], cholesterol [25], glucose [26], albumin [27], and total protein [28] were measured in the plasma, while globulin levels were derived by subtracting albumin from total protein. Direct radioimmunoassay was conducted on typical plasma samples to quantify hormone levels of triiodothyronine (T3), thyroxine (T4), progesterone (P4), and estradiol-17β (E2), utilizing Diagnostic Products Corporation kits (Los Angeles, USA) in accordance with the manufacturer's guidelines.

### Reproductive Parameters of She-Camels

Reproductive performance during the postpartum period was evaluated by recording several parameters [29]. Postpartum interval to first estrus (days) was monitored through daily observation of estrus-related behaviors, including signs of restlessness, increased aggression, and vulvar swelling accompanied by discharge. The first estrus was recorded as the day these behavioral signs were first detected following parturition. The number of services per conception was tracked by recording the number of mating attempts required until pregnancy was confirmed via ultrasound. Days open were calculated as the period from calving to successful conception, which was determined based on estrus detection and pregnancy confirmation. Placental drop was noted by observing the expulsion of the placenta following birth, while uterine involution was assessed through transrectal palpation and ultrasonography, monitoring the reduction in uterine size until complete involution was confirmed. The calving interval was determined as the number of days between two consecutive calving.

### Statistical Analysis

The collected data were analyzed using a completely randomized design to evaluate the effects of dietary levels of cobalt glucoheptonate supplementation. The analysis was performed using SAS software [30], applying the following statistical model: ${\text{Y}}_{\text{ij}}=\upmu +{\text{G}}_{\text{i}}+{\upvarepsilon }_{\text{ij}}$

Where: Y_ij_ ​represents the observed values; μ is the overall mean; G_i_​ refers to the effect of the experimental group, and ɛ_ij_​ is the random error. Differences among the experimental groups were assessed at a significance level of 5%, and Duncan’s multiple range test was used to determine specific group differences. Orthogonal contrasts were performed to evaluate the linear and quadratic responses to the incremental levels of dietary cobalt supplementation.

## Results

### Live Body Weight

**Fig. 1:**
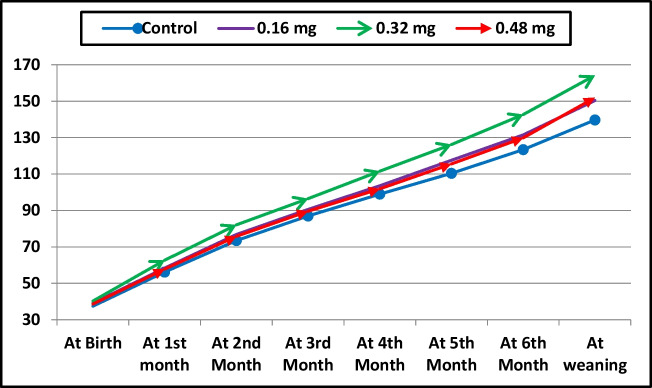
Calves weight from birth to weaning as affected with dietary cobalt supplementation

**Fig. 2:**
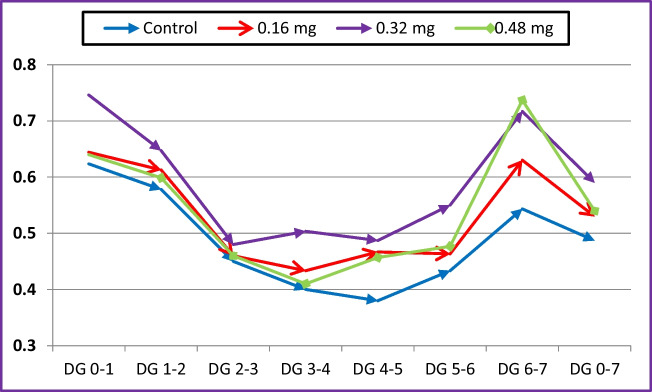
Calves daily weight gain from birth to weaning as affected with dietary cobalt supplementation

The results presented in Table 2 show that dietary cobalt supplementation had no significant effect on the live body weight of she-camels during the pre- and post-natal periods. Similarly, changes in she-camel weight over these periods were not significant. At calving, the weight of fetal fluids and the placenta was not significantly affected by cobalt treatment compared to the control group. Additionally, cobalt supplementation did not significantly affect the birth weight of camel calves. However, both the weaning weight and the average daily gain of calves were significantly affected by cobalt treatment (*P* < 0.001). These parameters exhibited significant linear (*P* = 0.001) and quadratic (*P* = 0.001) trends, with the highest values observed in calves whose dams were supplemented with 0.32 mg/kg cobalt. During the 7-week suckling period, calves born to dams receiving cobalt supplementation exhibited greater monthly body weight and weight gain compared to the control group (Figs. 1 and 2). The highest daily gain was recorded in calves from dams supplemented with 0.32 mg/kg cobalt.

**Table 2:** Effect of cobalt supplementation on growth performance of she-camels and newborn calves

	Dietary cobalt supplementation				*P*-value		
	0 mg/kg (Control)	0.16 mg/kg	0.32 mg/kg	0.48 mg/kg	ANOVA	Linear	Quadratic
She-camel weight before calving (kg)	487.0 ± 10.87	484.0 ± 5.07	484.0 ± 6.22	501.0 ± 3.83	0.297	0.200	0.174
She-camel weight after calving, kg	436.0 ± 7.97	430.5 ± 4.91	429.0 ± 4.81	448.5 ± 4.05	0.099	0.172	0.041
Weight of fetal fluids (kg)	10.44 ± 0.154	11.12 ± 0.291	11.22 ± 0.477	10.62 ± 0.402	0.357	0.690	0.088
Weight of placenta (kg)	3.12 ± 0.143	3.30 ± 0.071	3.52 ± 0.086	3.28 ± 0.183	0.221	0.241	0.122
Average weight of born calves							
At birth (kg)	37.40 ± 3.129	39.06 ± 2.174	40.22 ± 2.139	38.56 ± 4.667	0.938	0.750	0.611
At weaning (kg)	139.64 ± 2.631^c^	150.36 ± 2.849^b^	164.14 ± 2.704^a^	151.92 ± 2.551^b^	< 0.001	0.001	0.001
Average daily gain (kg)	0.487 ± 0.006^c^	0.530 ± 0.005^b^	0.590 ± 0.017^a^	0.540 ± 0.016^b^	< 0.001	0.001	0.001

Means in the same row with different superscripts are significantly different at *P* < 0.05. Values are represented as the mean ± standard errors

### Reproductive Performance

Dietary cobalt supplementation significantly influenced the reproductive performance of she-camels (Table 3). The postpartum interval to first estrus decreased linearly (*P* = 0.007) and quadratically (*P* < 0.001) with increasing cobalt levels, with the shortest interval observed in the 0.32 mg/kg group. The number of services per conception also decreased significantly (*P* = 0.002), showing both linear (*P* = 0.004) and quadratic (*P* = 0.003) trends, with all cobalt-supplemented groups requiring fewer services than the control. Days open were reduced linearly (*P* < 0.001) and quadratically (*P* = 0.015), with the lowest value recorded for the 0.48 mg/kg group. Placental drop time was affected quadratically (*P* = 0.011), with the 0.32 mg/kg group showing the shortest time, while the control group had the longest. Uterine involution time was significantly reduced (*P* < 0.001), showing both linear (*P* = 0.002) and quadratic (*P* < 0.001) effects, with the shortest duration observed in the 0.32 mg/kg group. The calving interval decreased linearly (*P* < 0.001), with the shortest interval recorded in the 0.48 mg/kg group.

**Table 3:** Effect of cobalt supplementation on reproductive performance of she-camels

	Dietary cobalt supplementation				*P*-value		
	0 mg/kg (Control)	0.16 mg/kg	0.32 mg/kg	0.48 mg/kg	ANOVA	Linear	Quadratic
Postpartum interval to 1st estrus (d)	67.20 ± 3.308^a^	52.00 ± 3.240^bc^	43.20 ± 2.223^c^	56.20 ± 3.089^b^	< 0.001	0.007	< 0.001
Number of services per conception	4.00 ± 0.316^a^	2.60 ± 0.245^b^	2.40 ± 0.245^b^	2.80 ± 0.200^b^	0.002	0.004	0.003
Days open (d)	277.00 ± 3.592^a^	225.00 ± 5.301^c^	255.00 ± 2.345^b^	180.00 ± 5.010^d^	< 0.001	< 0.001	0.015
Placental drop (min)	174.20 ± 3.056^a^	162.80 ± 3.308^ab^	155.60 ± 3.516^b^	165.80 ± 4.954^ab^	0.024	0.073	0.011
Uterine involution (d)	50.80 ± 2.083^a^	40.60 ± 1.327^bc^	36.60 ± 1.887^c^	43.20 ± 1.241^b^	< 0.001	0.002	< 0.001
Calving interval (d)	527.20 ± 3.089^a^	496.00 ± 5.831^b^	501.80 ± 7.889^b^	458.20 ± 3.967^c^	< 0.001	< 0.001	0.277

Means in the same row with different superscripts are significantly different at *P* < 0.05. Values are represented as the mean ± standard errors

### Milk Production and Composition

**Fig. 3:**
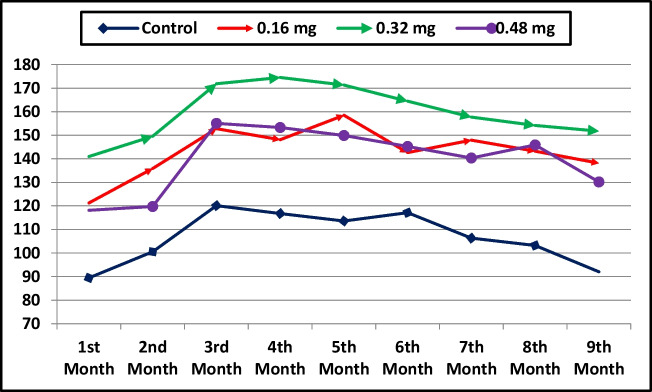
Monthly milk yield of She-camel as affected with dietary cobalt supplementation

Table 4 shows that cobalt supplementation in she-camel diets significantly affected daily milk yield, total milk yield, and lactation period (*P* < 0.001). Animals fed cobalt-supplemented diets produced higher total and monthly milk yields (Table 4, Fig. 3). The lactation period increased linearly (*P* = 0.004) and quadratically (*P* < 0.001), with the longest duration observed in the 0.32 mg/kg group. Total milk yield and daily milk yield also increased significantly (*P* < 0.001), with linear (*P* < 0.001) and quadratic (*P* < 0.001 for total yield, *P* = 0.019 for daily yield) trends, and the highest yields recorded at 0.32 mg/kg cobalt. Milk protein, lactose, total solids, and solids-not-fat yields were significantly influenced by cobalt supplementation. Protein yield showed linear (*P* = 0.030) and quadratic (*P* = 0.037) responses, with the greatest yield observed in the 0.32 mg/kg group. Lactose yield followed a linear (*P* = 0.014) and quadratic (*P* = 0.045) trend, with the highest value at 0.32 mg/kg cobalt. Total solids and solids-not-fat yields exhibited significant linear (*P* = 0.001, *P* = 0.002) and quadratic (*P* = 0.001, *P* = 0.005) effects, with maximum yields at 0.32 mg/kg. The cobalt concentration in milk increased linearly (*P* = 0.001), with higher levels recorded in all cobalt-supplemented groups compared to the control. However, cobalt supplementation had no significant effect on milk fat, protein, lactose, ash, total solids, or solids-not-fat percentages.

**Table 4:** Effect of cobalt supplementation on milk yield and milk composition of She-camels

	Dietary cobalt supplementation				*P*-value		
	0 mg/kg (Control)	0.16 mg/kg	0.32 mg/kg	0.48 mg/kg	ANOVA	Linear	Quadratic
Milk yield:							
Lactation period (d)	260.00 ± 4.909^c^	319.40 ± 5.653^b^	320.52 ± 8.404^a^	292.60 ± 6.90^b^	< 0.001	0.004	< 0.001
Total milk yield (kg)	959.04 ± 12.394^c^	1288.51 ± 22.681^b^	1436.43 ± 33.863^a^	1257.83 ± 7.180^b^	< 0.001	< 0.001	< 0.001
Daily milk yield (kg)	3.694 ± 0.088^c^	4.037 ± 0.072^b^	4.487 ± 0.098^a^	4.311 ± 0.130^ab^	< 0.001	< 0.001	0.019
Milk fat yield (kg)	36.035 ± 3.130	50.684 ± 5.407	60.211 ± 8.817	45.681 ± 8.036	0.123	0.220	0.046
Milk protein yield (kg)	30.184 ± 5.102^b^	43.684 ± 5.333^ab^	61.684 ± 7.287^a^	46.503 ± 7.134^ab^	0.022	0.030	0.037
Milk lactose yield (kg)	39.307 ± 4.687^b^	53.919 ± 5.711^ab^	66.807 ± 5.807^a^	57.601 ± 5.646^a^	0.020	0.014	0.045
Milk total solids yield (kg)	115.543 ± 7.871^c^	162.234 ± 8.179^b^	203.776 ± 16.597^a^	163.815 ± 6.963^b^	< 0.001	0.001	0.001
Milk solids not fat yield (kg)	79.508 ± 7.976^c^	111.550 ± 11.471^b^	143.565 ± 9.631^a^	118.134 ± 5.496^ab^	0.001	0.002	0.005
Milk composition:							
Fat %	3.764 ± 0.339	3.936 ± 0.417	4.156 ± 0.536	3.620 ± 0.617	0.879	0.924	0.480
Protein %	3.138 ± 0.523	3.386 ± 0.402	4.294 ± 0.490	3.706 ± 0.580	0.424	0.262	0.418
Lactose %	4.086 ± 0.463	4.194 ± 0.469	4.656 ± 0.393	4.570 ± 0.421	0.751	0.343	0.827
Ash %	1.044 ± 0.194	1.070 ± 0.200	1.048 ± 0.201	1.118 ± 0.203	0.993	0.825	0.914
Total solids %	12.032 ± 0.757	12.586 ± 0.578	14.154 ± 0.975	13.014 ± 0.482	0.240	0.182	0.258
Solids not-fat %	8.268 ± 0.790	8.650 ± 0.863	9.998 ± 0.627	9.394 ± 0.445	0.337	0.151	0.492
Cobalt (ppm)	0.970 ± 0.032^b^	1.1320 ± 0.042^a^	1.208 ± 0.047^a^	1.236 ± 0.056^a^	0.003	0.001	0.158

Means in the same row with different superscripts are significantly different at *P* < 0.05. Values are represented as the mean ± standard errors

### Immunoglobulins in Colostrum

Concentrations of immunoglobulins (IgG, IgM, and IgA) in the colostrum of she-camels during the first three postpartum days were significantly higher (*P* < 0.05) in all cobalt-supplemented groups compared to the control group (Table 5). IgA concentrations on the second day increased linearly (*P* = 0.026) and quadratically (*P* = 0.037), with higher values recorded in all cobalt-supplemented groups compared to the control. On the third day, a significant linear effect (*P* = 0.001) was observed, with the highest IgA concentration found in the 0.48 mg/kg group. On the second day, cobalt supplementation significantly increased IgM levels (*P* = 0.009), with a linear (*P* = 0.006) and quadratic (*P* = 0.042) response. The third day showed a significant quadratic effect (*P* = 0.070), with higher IgM levels in the supplemented groups. IgG concentrations increased significantly (*P* < 0.001) across all days. A strong linear (*P* < 0.001) and quadratic (*P* < 0.001) effect was noted, with the 0.32 mg/kg group consistently showing the highest IgG levels.

**Table 5:** Effect of cobalt supplementation on colostrum immunoglobulins concentrations of she-camels on the 1st three postpartum days

	Dietary cobalt supplementation				*P*-value		
	0 mg/kg (Control)	0.16 mg/kg	0.32 mg/kg	0.48 mg/kg	ANOVA	Linear	Quadratic
IgA (mg/ml)							
1st day	2.760 ± 0.198	3.540 ± 0.363	3.820 ± 0.405	3.860 ± 0.220	0.080	0.020	0.250
2nd day	2.900 ± 0.252^b^	3.700 ± 0.259^a^	3.960 ± 0.214^a^	3.680 ± 0.222^a^	0.033	0.026	0.037
3rd day	2.300 ± 0.168^b^	3.260 ± 0.172^a^	3.320 ± 0.294^a^	3.740 ± 0.298^a^	0.005	0.001	0.280
IgM (mg/ml)							
1st day	2.960 ± 0.285	3.780 ± 0.442	4.220 ± 0.201	3.940 ± 0.244	0.056	0.026	0.092
2nd day	2.820 ± 0.269^b^	4.020 ± 0.242^a^	3.960 ± 0.236^a^	4.040 ± 0.266^a^	0.009	0.006	0.042
3rd day	3.020 ± 0.280^b^	4.200 ± 0.217^a^	3.880 ± 0.357^a^	3.960 ± 0.262^a^	0.048	0.066	0.070
IgG (mg/ml)							
1st day	32.800 ± 0.860^c^	44.800 ± 1.241^b^	49.200 ± 0.917^a^	42.000 ± 0.707^b^	< 0.001	< 0.001	< 0.001
2nd day	32.000 ± 0.707^c^	42.400 ± 1.030^b^	46.600 ± 0.812^a^	45.000 ± 1.643^ab^	< 0.001	< 0.001	< 0.001
3rd day	27.800 ± 0.583^c^	40.200 ± 1.158^ab^	42.000 ± 1.049^a^	37.400 ± 1.249^b^	< 0.001	< 0.001	< 0.001

Means in the same row with different superscripts are significantly different at *P* < 0.05. Values are represented as the mean ± standard errors

### Blood Biochemical Parameters

The effects of dietary cobalt treatment on blood parameters during the pre- and post-natal periods are presented in Table 6. One month pre-partum, cobalt supplementation significantly affected total protein levels (*P* < 0.001), with the 0.32 mg/kg group recording the highest values. Albumin levels showed both linear (*P* = 0.001) and quadratic (*P* < 0.001) effects, with the 0.32 mg/kg group achieving the highest concentration. Also, cobalt supplementation significantly increased glucose (*P* = 0.011), triglycerides (*P* = 0.001), and cobalt concentrations (*P* < 0.001). The 0.32 mg/kg group demonstrated the highest values for both cobalt and triglycerides compared to the other groups. Post-partum, cobalt supplementation continued to show significant effects on total proteins (*P* = 0.011) and albumin (*P* = 0.010), with the 0.32 mg/kg group demonstrating the highest concentrations for both. Cobalt levels also significantly increased (*P* < 0.001) after supplementation, with the 0.32 mg/kg group showing the highest levels. The globulin concentrations were significantly affected by cobalt supplementation (*P* = 0.032), with the 0.32 mg/kg group having the highest levels. Additionally, B_12_ concentrations were significantly influenced by cobalt supplementation during both pre- and post-partum periods (*P* = 0.003 and *P* = 0.017, respectively), with higher B_12_ levels in the supplemented groups compared to the control.

**Table 6:** Effect of cobalt supplementation on pre- and post-partum concentrations of some biochemical in blood plasma of lactating she-camels

	Dietary cobalt supplementation				*P*-value		
	0 mg/kg (Control)	0.16 mg/kg	0.32 mg/kg	0.48 mg/kg	ANOVA	Linear	Quadratic
One month pre-partum:							
Total proteins (g/dl)	5.426 ± 0.090^b^	6.890 ± 0.276^a^	7.770 ± 0.357^a^	6.866 ± 0.365^a^	< 0.001	0.001	0.001
Albumin (g/dl)	3.030 ± 0.231^c^	4.176 ± 0.343^ab^	4.472 ± 0.115^a^	3.546 ± 0.185^bc^	0.002	0.097	< 0.001
Globulin (g/dl)	2.396 ± 0.250	2.714 ± 0.171	3.298 ± 0.329	3.320 ± 0.331	0.083	0.016	0.602
Glucose (mg/dl)	47.740 ± 0.665^b^	55.174 ± 1.008^a^	52.742 ± 0.946^a^	53.434 ± 1.673^a^	0.002	0.011	0.009
Cholesterol (mg/dl)	84.540 ± 2.859	81.800 ± 3.536	75.840 ± 2.233	82.690 ± 2.129	0.175	0.363	0.100
Triglycerides (mg/dl)	99.700 ± 1.439^b^	107.020 ± 1.523^a^	107.580 ± 0.408^a^	104.200 ± 1.077^a^	0.001	0.018	< 0.001
Cobalt (µg/l)	0.450 ± 0.027^b^	0.972 ± 0.037^a^	1.056 ± 0.010^a^	1.000 ± 0.035^a^	< 0.001	< 0.001	< 0.001
Iron (µg/l)	0.736 ± 0.048	0.674 ± 0.025	0.748 ± 0.072	0.678 ± 0.020	0.571	0.636	0.932
B_12_ (µg/l)	6.442 ± 0.185^b^	8.066 ± 0.529^a^	8.706 ± 0.385^a^	8.246 ± 0.306^a^	0.003	0.002	0.013
One month postpartum:							
Total proteins (g/dl)	5.888 ± 0.173^b^	7.398 ± 0.510^a^	7.7180 ± 0.369^a^	7.348 ± 0.304^a^	0.011	0.010	0.019
Albumin (g/dl)	3.494 ± 0.124^b^	4.098 ± 0.296^ab^	4.7800 ± 0.182^a^	4.160 ± 0.265^ab^	0.010	0.018	0.016
Globulin (g/dl)	2.394 ± 0.202^b^	3.300 ± 0.254^a^	2.938 ± 0.191^ab^	3.188 ± 0.081^a^	0.020	0.032	0.108
Glucose (mg/dl)	58.200 ± 3.878	67.400 ± 2.768	68.400 ± 2.135	69.000 ± 3.564	0.090	0.031	0.193
Cholesterol (mg/dl)	81.800 ± 2.746	87.400 ± 3.723	87.800 ± 1.685	83.800 ± 2.939	0.410	0.624	0.114
Triglycerides (mg/dl)	99.800 ± 1.594	103.400 ± 1.720	102.000 ± 2.302	101.200 ± 1.241	0.546	0.726	0.228
Cobalt (µg/l)	0.492 ± 0.034^b^	0.754 ± 0.029^a^	0.802 ± 0.032^a^	0.744 ± 0.034^a^	< 0.001	< 0.001	< 0.001
Iron (µg/l)	0.416 ± 0.060	0.450 ± 0.032	0.426 ± 0.040	0.428 ± 0.035	0.953	0.951	0.716
B_12_ (µg/l)	6.850 ± 0.315^b^	8.494 ± 0.435^a^	8.258 ± 0.311^a^	8.0240 ± 0.148^a^	0.017	0.048	0.015

Means in the same row with different superscripts are significantly different at *P* < 0.05. Values are represented as the mean ± standard errors

### Hormonal Parameters

The effects of dietary treatment on the concentrations of thyroid and ovarian hormones in she-camels during the pre- and post- partum periods are presented in Table 7. Pre-partum, cobalt supplementation significantly affected (*P* < 0.001), with the 0.32 mg/kg and 0.48 mg/kg groups exhibiting higher concentrations compared to the control. Similarly, T4 levels were significantly higher in the supplemented groups (*P* < 0.001), with the 0.32 mg/kg group having the highest values. The P4 concentrations also showed a significant difference (*P* = 0.015), with the 0.48 mg/kg group having the highest levels. Also, E2 concentration was significantly influenced by cobalt supplementation (*P* < 0.001), with the 0.32 mg/kg and 0.48 mg/kg groups showing higher concentrations compared to the control. Post-partum, T3 levels remained significantly higher in the supplemented groups (*P* < 0.001), with no significant differences between the 0.32 mg/kg and 0.48 mg/kg groups. T4 concentrations continued to show a significant increase (*P* = 0.002) in the supplemented groups. Also, E2 (*P* = 0.007) concentrations were significantly elevated (*P* = 0.007), with the 0.32 mg/kg and 0.48 mg/kg groups demonstrating higher values than the control.

**Table 7:** Effect of cobalt supplementation on plasma concentration of thyroid and ovarian hormones of she-camels

	Dietary cobalt supplementation				*P*-value		
	0 mg/kg (Control)	0.16 mg/kg	0.32 mg/kg	0.48 mg/kg	ANOVA	Linear	Quadratic
Triiodothyronine (T_3_; πg/dl)							
Pre-partum	86.790 ± 2.480^b^	104.600 ± 3.027^a^	111.620 ± 2.356^a^	112.740 ± 3.344^a^	< 0.001	< 0.001	0.009
Post-partum	89.600 ± 1.806^b^	103.600 ± 1.691^a^	103.000 ± 1.517^a^	102.100 ± 0.812^a^	< 0.001	< 0.001	< 0.001
Thyroxine (T_4_; πg/dl)							
Pre-partum	1.520 ± 0.137^b^	3.000 ± 0.303^a^	3.280 ± 0.156^a^	3.230 ± 0.290^a^	< 0.001	< 0.001	0.005
Post-partum	2.470 ± 0.115^b^	3.060 ± 0.117^a^	3.160 ± 0.204^a^	3.400 ± 0.100^a^	0.002	< 0.001	0.229
Progesterone (P_4_; πg/dl)							
Pre-partum	2.702 ± 0.126^b^	3.662 ± 0.198^a^	3.576 ± 0.259^a^	3.902 ± 0.333^a^	0.015	0.005	0.208
Post-partum	2.408 ± 0.156	2.666 ± 0.125	2.922 ± 0.167	2.968 ± 0.257	0.154	0.031	0.570
Estradiol (E_2_; pg/dl)							
Pre-partum	39.330 ± 1.549^b^	75.440 ± 2.572^a^	73.300 ± 2.618^a^	77.570 ± 1.900^a^	< 0.001	< 0.001	< 0.001
Post-partum	40.400 ± 2.205^b^	46.332 ± 3.159^ab^	53.600 ± 1.913^a^	51.100 ± 2.205^a^	0.007	0.002	0.100

Means in the same row with different superscripts are significantly different at *P* < 0.05. Values are represented as the mean ± standard errors

## Discussion

In the current study, calf body weight and daily gain increased quadratically with different cobalt supplementation levels, with the highest values observed in the group supplemented with 0.32 mg cobalt. These results are contradictory to those of Tiffany et al. [31] who reported better average daily gain and gain efficiency in cobalt supplemented steers receiving a 0.04 to 0.05 mg/kg diet. Similarly, Bishehsari et al. [32] identified enhanced growth performance metrics in lambs supplemented with cobalt, which were fed a basal diet comprising 0.088 ppm cobalt. Johnson et al. [33] observed enhanced growth rates in goats supplemented with cobalt, receiving a diet that included 0.1 mg cobalt/kg diet. Also, Dezfoulian and Aliarabi [34] observed that administering higher quantities of cobalt (0.25 *vs*. 0.5 mg/kg dry matter) enhanced feed consumption and growth rate in kid goats. The improved performance observed in newborn calves in our study can be attributed to cobalt supplementation in their mothers during the three months prepartum. Cobalt plays a crucial role in enhancing nutrient digestibility through its impact on rumen microbial populations and vitamin B_12_ synthesis [34, 35]. This improved digestion likely contributed to better nutrient transfer to the calves during gestation, resulting in enhanced growth and performance post-birth. Additionally, vitamin B_12_-dependent enzymes, essential for protein and energy metabolism, likely supported this improved performance [36]. Of note, the lower dietary concentration of cobalt (0.32 mg/kg diet) has been shown to enhance growth performance in calves compared to a higher dose (0.48 mg/kg diet), likely due to improved nutrient utilization and metabolic efficiency. Excessive cobalt levels may interfere with the absorption of other essential minerals, such as iron and zinc, thereby negatively affecting growth. Previous research has suggested that the optimal cobalt supply for maximum growth in cattle is approximately 0.20 mg/kg dietary dry matter [37]. Lower cobalt concentrations have also been shown to improve nutrient digestibility, with ultrafine cobalt particles increasing dry matter digestibility by 9% at lower doses [38]. Moreover, studies have demonstrated that calves receiving lower dietary cobalt levels exhibit better feed conversion ratios and daily weight gains compared to those on higher cobalt diets [39, 40].

The inclusion of cobalt in the diets of She-camels enhanced their reproductive performance during lactation. This was evident through a reduction in the time to the first heat onset, fewer number of services per conception, shorter days open, quicker placental drop, faster uterine involution, and a decreased calving interval, alongside an increased conception rate. The improvement in reproductive performance can be linked to the essential roles cobalt plays in vitamin B_12_ synthesis and overall metabolic processes [15, 41]. Cobalt is critical for rumen microorganisms, which produce vitamin B_12_, a key cofactor in numerous metabolic pathways which requires for efficient protein and energy metabolism, particularly during the transition from pregnancy to early lactation [42]. Adequate energy and protein metabolism are directly related to reproductive health and efficiency. In a study conducted by El Sayed et al. [43], it was observed that cobalt supplementation resulted in a shorter postpartum first estrous interval and fewer services per conception in cows receiving higher cobalt levels (0.57 ppm), leading to improved conception rates of 80% within 120 days postpartum. It was reported also that cobalt deficiency correlated with a heightened occurrence of quiet estrus, a postponed onset of puberty, non-functional ovaries, and abortion. Insufficient dietary cobalt levels have been associated with heightened early calf mortality [44].

The transition from late gestation to lactation in she-camels involves significant metabolic adaptations. The increased demand for glucose is crucial for fetal growth in late pregnancy and for lactose synthesis in early lactation. Vitamin B_12_ plays an essential role in glucose production from propionate, acting as a co-enzyme for methylmalonyl-CoA mutase [41, 45]. A deficiency in vitamin B_12_ can result in reduced milk synthesis [46]. Furthermore, methionine, a key amino acid for milk production, is regenerated through the activity of methionine synthase, an enzyme dependent on vitamin B_12_ [47]. Adequate B_12_ levels are thus critical for both glucose metabolism and efficient milk production in lactating she-camels.

In the present study, cobalt supplementation in the diets of she-camels led to an increase in milk yield. Specifically, camels receiving 0.32 mg cobalt/kg diet produced more milk and had a longer lactation period compared to those without cobalt supplementation. Milk protein, lactose, milk fat, total solids and solids not fat yield increased as cobalt supplementation in She-camel diets. She-camel group fed diet supplemented with 0.32 mg cobalt/kg diet recorded higher milk yield of protein, lactose, total solids and solids not fat. These findings align with Girard and Matte [48], who reported increased milk production in cows injected with vitamin B_12_, highlighting the role of cobalt in B_12_ synthesis. Similarly, Kincaid et al. [49] observed a significant interaction between time, treatment, and parity, where multiparous cows showed higher milk yields in response to cobalt supplementation. Akins et al. [50] indicated that a deficiency of cobalt and vitamin B_12_ after parturition can lead to reduced milk production and diminished quantity and quality of colostrum. Nevertheless, certain studies indicated no substantial impact of dietary cobalt supplementation on milk yield in cows, implying that diets with 0.19 to 0.93 mg cobalt/kg may not affect milk production [43]. This variability in results suggests that the effectiveness of cobalt supplementation may depend on factors such as dosage, species, dietary form, and lactation stage.

Notably, in our study, a higher concentration of cobalt (0.48 mg) resulted in lower lactation performance compared to the 0.32 mg concentration. Cobalt is an essential trace element primarily required for the synthesis of vitamin B_12_ by rumen microbes. Vitamin B_12_ plays a crucial role in metabolic pathways, including carbohydrate and lipid metabolism, which are critical for energy production and milk synthesis [15]. However, excessive dietary cobalt can lead to nutrient imbalances, particularly by interfering with the absorption and utilization of other vital nutrients [51]. Furthermore, an excessive cobalt supply without appropriate balance with nutrients such as phosphorus may fail to support optimal milk yield and could even result in adverse effects [52].

Herein, cobalt supplementation significantly increased the yields of protein, lactose, total solids, and solids not fat, but did not alter milk composition, including lactose, protein, fat, and solids-not-fat percentages. The increased yields reflect an improvement in milk production and lactation efficiency, rather than alterations in the concentrations of the milk's components. These findings are consistent with those of Kincaid et al. [49], who reported no changes in the fat and protein content of milk following dietary cobalt treatment. Similarly, Kincaid and Socha [53] observed no effect of cobalt supplementation on milk composition, including fat and protein percentages and yields. In line with these results, Croom Jr et al. [54] found that weekly injections of 15 mg of vitamin B_12_ during the early lactation period did not influence milk fat percentage.

Cobalt content in milk increased in she-camels supplemented with cobalt compared to the control group, with a positive correlation between cobalt supplementation levels and milk cobalt concentration. Kincaid and Socha [53] similarly observed that dietary cobalt supplementation increased vitamin B_12_ concentrations in colostrum and milk. Lopez-Guisa and Satter [55] suggested that cobalt supplementation enhances dry matter degradation by rumen microbes, potentially due to cobalt’s role as a bridge between the negatively charged surfaces of plant and bacterial cell walls. Another possibility is that cobalt is required in higher concentrations than previously thought for optimal microbial growth. Although cobalt supplementation beyond NRC recommendations has been shown to improve organic matter digestibility, animal performance was only slightly affected. Kincaid and Socha [53] also noted that vitamin B_12_ levels tend to decrease during the early dry period, and adding cobalt to the diet may enhance ruminal vitamin B_12_ synthesis, leading to increased concentrations in colostrum and milk. In the current study, colostrum immunoglobulin concentrations in she-camels during the first three days postpartum increased with dietary cobalt supplementation. Similar findings have been observed in cows, where cobalt supplementation positively influenced the immunoglobulin profile in colostrum, which is critical for calf health. Cobalt plays an essential role in the production of vitamin B_12_, a vital cofactor for B-cell proliferation and antibody production [56]. This enhanced vitamin B_12_ availability likely supports more robust immune responses, leading to higher immunoglobulin concentrations in colostrum.

The observed increase in serum total proteins, albumin, globulin, glucose, total cholesterol, and triglycerides in she-camels fed cobalt-supplemented diets compared to those without supplementation can be linked to the metabolic role of cobalt in enhancing vitamin B_12_ synthesis, which is essential for protein metabolism and energy regulation [47]. Vitamin B_12_ plays a critical role in protein and lipid metabolism, likely contributing to the elevated concentrations of these biochemical markers [15]. The increase in serum cobalt and vitamin B_12_ concentrations, particularly in she-camels fed diets supplemented with 0.32 mg cobalt/kg, aligns with studies in ruminants that show dietary cobalt supplementation enhances cobalt absorption and vitamin B_12_ synthesis by rumen microbes [53]. This effect is crucial for improving energy metabolism, as vitamin B_12_ acts as a cofactor for enzymes like methylmalonyl-CoA mutase, which is essential for propionate conversion to glucose [41, 45].

Increased concentrations of T4, T3, progesterone, and estradiol in the blood serum of cobalt-supplemented she-camels further highlight cobalt's role in regulating metabolic and reproductive functions. Vitamin B_12_ plays a role in the conversion of homocysteine to methionine, a process that is essential for the production of S-adenosylmethionine (SAMe) [15, 57]. SAMe is involved in the methylation of thyroid hormones in the liver and other tissues [58]. The elevated levels of reproductive hormones, specifically P4 and E2 detected in the current study, may indicate improved ovarian function. Studies have shown that vitamin B_12_ and folic acid increase follicular fluid volume and promote earlier cell differentiation in dominant follicles, suggesting enhanced ovarian function [59]. Additionally, improved energy metabolism in the postpartum period can support better reproductive outcomes and may influence P4 and E2 levels [59]. Elevated estrogen levels during estrus are associated with higher conception rates, indicating that vitamin B_12_ may indirectly boost estrogen production by supporting ovarian activity [60].

## Conclusion

The results indicate that dietary cobalt supplementation at 0.32 mg/kg from three months prepartum to nine months postpartum significantly improved lactation performance, reproductive efficiency, blood biochemical parameters, and colostrum immunoglobulin levels in Maghrabi she-camels. Additionally, it enhanced the growth performance of their calves. Future research should focus on understanding the mechanisms behind these improvements and the long-term impacts of cobalt supplementation on camel health and productivity.

## Data Availability

The data that support the findings of this study are available from the corresponding author upon reasonable request.
